# Establishment and validation of a ferret model for systemic antibiotic treatment during influenza A virus infection

**DOI:** 10.1038/s41684-025-01574-9

**Published:** 2025-06-19

**Authors:** Eric M. Velazquez, Poulami Basu Thakur, Nicole Brock, Taronna R. Maines, Jessica A. Belser

**Affiliations:** 1 Comparative Medicine Branch, Division of Core Laboratory Services and Response, Centers for Disease Control and Prevention, Atlanta, GA USA; 2 Immunology and Pathogenesis Branch, Influenza Division, Centers for Disease Control and Prevention, Atlanta, GA USA; 3 Graduate Division of Biological and Biomedical Sciences, Emory University, Atlanta, GA USA

**Keywords:** Influenza virus, Model vertebrates, Antibiotics

## Abstract

The ferret has been widely used to study both the pathogenicity and the transmissibility of respiratory viral infections, but little is known about how host-associated microbial communities alter disease susceptibility owing to the lack of a validated model. Here, we compared the safety of injectable versus oral broad-spectrum antibiotics and their efficacy in reducing culturable bacteria from the upper respiratory tract of ferrets before an influenza A virus challenge. Both injectable and oral antibiotic treatment led to drastic reductions in cultivable bacteria from nasal wash specimens when assessed after 7 days of ongoing treatment. Even when extended to 14 days, there were few adverse events noted and no clinically significant bloodwork changes. During challenge with either a high-dose or low-dose A(H1N1)pdm09 influenza A virus inoculum, all animals became productively infected and had generally similar viral titers and clinical measurements, regardless of antibiotic pretreatment. Collectively, these results support that both antibiotic regimens evaluated in laboratory ferrets can be utilized to further characterize host–microbial interactions in the context of respiratory viral infections and other pathogens, including a needle-free approach that may be suitable for studies of high-consequence pathogens in containment laboratory facilities.

## Abstract (web summary)

The authors compare the efficacy of injectable and oral broad-spectrum antibiotics on reducing bacteria from the upper respiratory tract of ferrets, before evaluating the effects of commensal bacteria reduction on influenza A virus infection.

## Main

The ferret (*Mustela putorius furo*) is underrepresented in laboratory settings but has been increasingly used for a variety of infectious and noninfectious disease models^1,2^. Importantly, the ferret is a preferred species in which to probe the pandemic potential of emerging influenza A viruses (IAVs)^3,4^, which continue to be associated with a substantial public health burden and an estimated 9–40 million human cases each year in the USA^5^. A notable strength of the ferret is its ability to recapitulate numerous clinical signs of infection observed in people^3^ owing to its similar physiological structure and features compared with the human respiratory tract. Given that disease severity after IAV infection in humans ranges from mild to severe, depending on the virus strain, host immune status and other parameters^6^, many of these determinants can be manipulated during experimental inoculation of animals in a controlled setting. In particular, the ferret is well suited to study pathogen coinfection scenarios in the context of both pathogenesis and transmission^7,8^; prior work using this host species has supported that primary influenza virus infection followed by a secondary challenge with opportunistic bacterial pathogens can lead to increased correlates of clinical disease with or without higher viral loads^9^. However, there are few studies in ferrets that have directly tested the role of host-associated microbial communities on disease susceptibility or severity during IAV challenge.

Microbial communities present in the mammalian upper respiratory tract (URT) are shaped by host and environmental factors, but compositional changes can occur during several disease states, including viral infections^10^. For example, nasopharyngeal samples from human patients have shown heightened abundance of certain microbial populations based on IAV-infection status and disease severity^11–13^. Notably, nasal wash (NW) specimens from IAV-challenged ferrets can show similar modulations in microbial populations during the acute phase of infection^13^. Given that IAV infection can modulate the ferret URT microbiome, it is important to distinguish whether perturbations of resident bacteria can alter disease outcomes. One study utilized topical mupirocin in ferrets to reduce local populations of bacteria from the nasal cavity before IAV inoculation; no differences were observed in viral titers or pathogenicity between treated and control animals^14^. However, use of this antibiotic ointment results in the selective reduction of mostly Gram-positive bacteria and few Gram-negative or anaerobic bacteria^15^. Furthermore, studies in the ferret model have used a high IAV challenge dose^13,14^, precluding evaluation of viral–bacterial interactions under more physiologically relevant conditions. Although these studies support the importance of examining IAV infection in the context of reduction of commensal bacteria, ferret models with systemic antibiotic depletion still need to be established to perform further investigations.

Repeat drug administration can be challenging in small animals and presents an additional source of stress to laboratory-housed species. Therefore, treatment protocols should be rationally selected on the basis of their safety data, if available. So far, there has not been a methodical examination comparing different antibiotic treatment modalities for efficacy in reducing culturable bacteria in the upper airways of ferrets. Fulfilling this prerequisite of simplifying a diverse microbiome would pave the way for future studies to further dissect specific relationships that may have a role in shaping disease outcomes. Furthermore, while some IAV must be manipulated in BSL3-enhanced laboratory facilities where reduced needle use is an important consideration, a comparative assessment of efficacy between injectable and oral administration of antibiotics in this species would be valuable to determine if needle-free approaches are feasible in these settings. To improve our ability to assess within-host viral–bacterial interactions, we first established a ferret model that lacked cultivable bacteria in the URT using two different antibiotic regimens and assessing their safety and efficacy profiles. Using this model, we next challenged antibiotic-treated or control animals with two different doses of a contemporary A(H1N1)pdm09 IAV to investigate whether the presence or absence of cultivable bacteria at sites of IAV replication can modulate clinical and virological parameters of infection.

## Results

### Robust reduction of cultivable bacteria in the ferret URT after antibiotic treatment

We first assessed the capacity of systemically administered antibiotics to deplete commensal bacteria in the ferret URT. Different antibiotic classes were chosen to achieve a broad spectrum of antimicrobial activity (specified in Table 1), administered once daily for 14 days by either repeated subcutaneous (SQ) injection or repeated oral syringe feedings (per os, PO), to assess the feasibility of multiple delivery approaches that may be most appropriate in different laboratory settings compared with mock-treated animals. To evaluate antibiotic treatment efficacy, a traditional culture-based approach was selected to quantify the remaining viable and potentially metabolically active fraction of the microbiome^16^; this method is sensitive to low-abundance organisms that may be present in upper airway specimens^17^.

**Table 1:** Antibiotic treatments administered to ferrets

Route^a^	Generic name	Class	Dose (mg/kg)	Volume (mL) administered^b^
SQ	Metronidazole	Nitroimidazole	20	4
SQ	Enrofloxacin	Fluoroquinolone	10	0.44
SQ	Piperacillin–tazobactam	β-Lactam	4	0.04
PO	Metronidazole	Nitroimidazole	20	0.33
PO	Enrofloxacin	Fluoroquinolone	10	0.5
PO	Amoxicillin–clavulanate acid	β-Lactam	20	0.4

^a^Ferrets were administered antibiotics daily by the SQ or PO route. Mock animals (not shown) received SQ injections with sterile saline and syringe feedings with only meat-flavored baby food. ^b^Volume specified per 1 kg ferret weight.

**Fig. 1:**
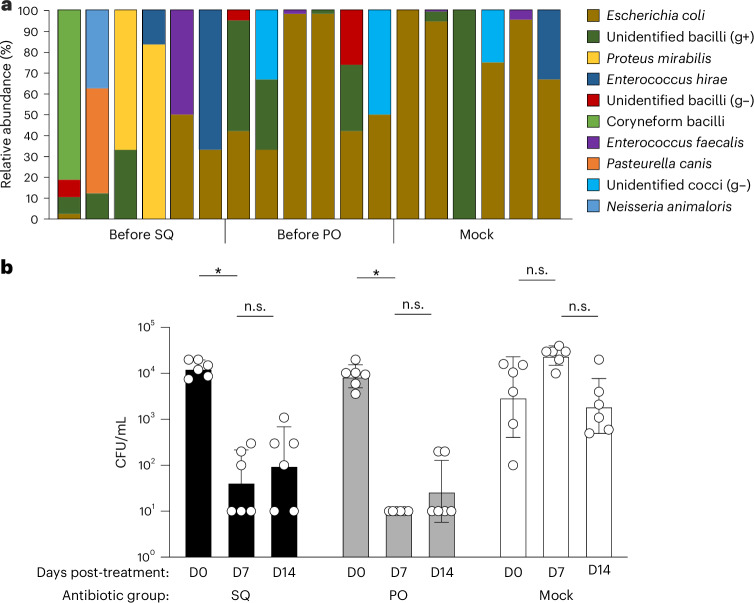
Efficacy and clinical tolerance of sustained antibiotic treatment in ferrets. NW specimens were collected for aerobic bacterial culture before first antibiotic treatment **a**, Relative abundances of the top ten taxa identified by MALDI-TOF were normalized per ferret. Gram-positive and Gram-negative are abbreviated as g+ and g–, respectively. **b**, Corresponding bacterial loads are displayed as geometric means ± s.d. (day (D)0). Ferrets then received injectable antibiotics (SQ), oral antibiotics (PO) or mock treatment, and bacterial loads were assessed at the indicated time points (D0, D7 and D14). Changes in bacterial loads were analyzed by multiple comparisons after repeated-measures two-way ANOVA. Bars represent geometric mean with geometric s.d. from day 0, 7 or 14 post-treatment. *n* = 6 ferrets per group. Limit of detection, 10 CFU. n.s., not significant. *P ≤ 0.05. Source data

NW specimens collected before antibiotic treatment showed similar bacterial colonization levels across ferrets in all groups when cultured aerobically (10^3^–10^4^ colony-forming units (CFU)/mL), or anaerobically, with variations in aerobic taxonomic groups present across different ferrets, in agreement with prior work^13^ (Fig. 1). Compared with baseline samples, levels of culturable aerobic bacteria in ferret NW specimens were reduced 100–1,000-fold (SQ group) or to undetectable levels (PO group) after 7 days of treatment (Fig. 1b). Reductions persisted through day 14 of dosing in both treatment groups. These results support that 7 days of either oral or injectable delivery of antibiotics could reliably reduce culturable aerobic bacteria in the URT of ferrets by several orders of magnitude.

### High clinical tolerance of sustained antibiotic treatment in ferrets

To monitor for potential antibiotic toxicity, we performed regular clinical assessments before, during and after the scheduled 14-day treatment. Overall, relatively few adverse effects were noted when each drug was administered in succession. One animal from the injection group developed minor superficial skin irritation with scabbing after 10 days at a single injection site, which resolved by the study endpoint. Two animals from the oral group developed localized gingival inflammation that resolved after 1–2 days without discontinuing antibiotic treatment. No evidence of aspiration was noted. As expected, oral dosing of metronidazole was met with strong resistance due to its bitter taste, despite mixing with meat-flavored baby food^18^. Because metronidazole is acidic, SQ injection caused more discomfort in those animals, possibly due to larger volume. No dehydration, weight loss, lethargy, changes in temperature or, remarkably, diarrhea was noted in any animal after 14 days (data not shown).

**Fig. 2:**
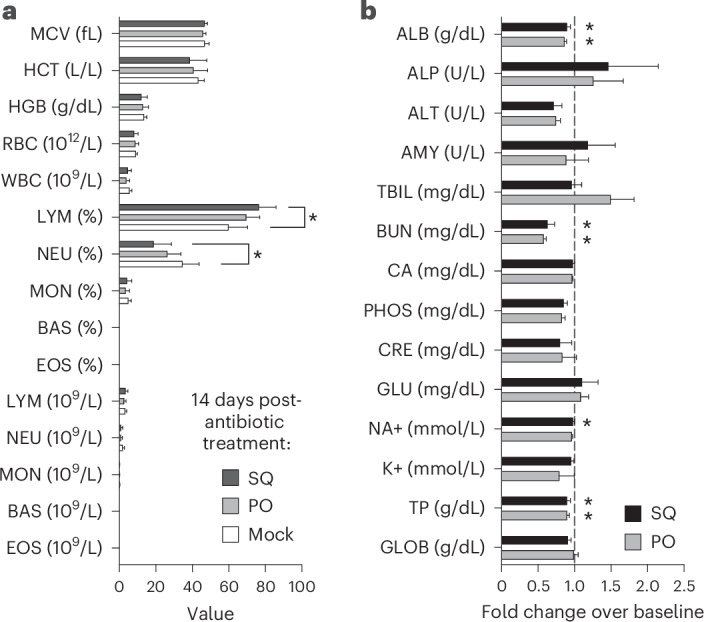
Serum chemistry and electrolyte testing during antibiotic treatment. Whole blood or serum was collected from each animal before first antibiotic treatment and analyzed as described in the Methods. Ferrets then received either oral (PO) or injectable (SQ) antibiotics for 14 days when a subsequent sample was obtained and analyzed. **a**, A comparison of complete blood count parameters between SQ, PO and mock-treated cohort animals 14 days after antibiotic treatment. BAS, basophil; EOS, eosinophils; HCT, hematocrit; HGB, hemoglobin; MCV, mean corpuscular volume; RBC, red blood cell. **b**, Linear fold changes in SQ and PO animals at day 14 after antibiotic treatment for each blood chemistry parameter listed compared with baseline samples. All levels are reported as the mean ± standard deviation of *n* = 6 animals per group. Parameters in **b** are represented as the day 14 value fold change over the pretreatment baseline (indicated with a vertical dashed line). Individual values and abbreviations in **b** are reported in Supplementary Table 1. Data were analyzed by Student’s *t*-test, *P < 0.05. Source data

Beyond observations of morbidity, we also examined the potential impact of sustained antibiotic treatment on lymphohematopoietic and electrolyte parameters in ferrets during the treatment period. Total white blood cell (WBC; Table 2) and red blood cell counts were generally stably maintained throughout the treatment period in all groups (Fig. 2a). Serum chemistry parameters were generally maintained at pretreatment levels after 14-day antibiotic administration in both groups (Supplementary Table 1); a few markers shifted in the absence of clinical correlates of disease (Fig. 2b and Supplementary Table 1). Collectively, these data support that both injectable and oral deliveries were well tolerated by ferrets during a 14-day administration.

**Table 2:** Complete blood count of ferrets receiving different antibiotic treatments

Cell type^a^		Treatment route		
	Days after treatment	Oral	Inject	Mock
WBC	0	6.30 ± 1.64	6.58 ± 2.66	8.21 ± 1.22
	7	3.79 ± 1.24	6.29 ± 1.35	4.59 ± 2.37
	14	4.07 ± 1.64	4.79 ± 1.88	5.78 ± 1.20
LYM	0	4.10 ± 0.89	4.28 ± 2.15	4.86 ± 0.94
	7	2.35 ± 0.73	4.42 ± 0.97	2.87 ± 1.65
	14	2.81 ± 0.93	3.54 ± 1.15	3.41 ± 0.71
MON	0	0.29 ± 0.12	0.30 ± 0.29	0.30 ± 0.26
	7	0.19 ± 0.80	0.31 ± 0.09	0.21 ± 0.10
	14	0.15 ± 0.11	0.20 ± 0.12	0.30 ± 0.11
NEU	0	1.91 ± 0.75	2.01 ± 0.82	3.04 ± 0.90
	7	1.25 ± 0.62	1.57 ± 0.52	1.51 ± 1.06
	14	1.17 ± 0.62	1.08 ± 0.76	2.06 ± 0.72

^a^WBC, total white blood cells; LYM, total lymphocytes; MON, total monocytes; NEU, total neutrophils. All counts are expressed as 10^9^/L and are reported as the mean ± s.d. of *n* = 6 animals per group.

### Comparable clinical course of IAV infection in ferrets during antibiotic treatment

Once established that a 14-day antibiotic treatment regimen was both efficacious and well tolerated by ferrets, we next assessed if treatment with multimodal antibiotics altered the course of clinical disease during IAV infection in ferrets. Animals treated with either injectable or oral antibiotics were challenged with either a high (10^6^ plaque-forming units (PFU) per animal) or low (10^3^ PFU per animal) dose of a contemporary A(H1N1)pdm09 IAV strain, A/Nebraska/14/2019 (Neb/14), on day 8 post-treatment, with treatment persisting through day 6 post-inoculation (p.i.) (14 days of treatment total).

**Fig. 3:**
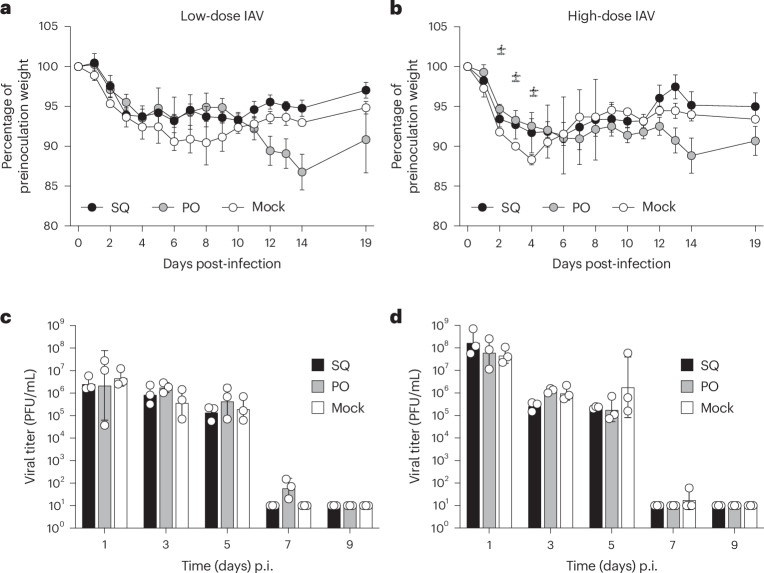
Weight loss and viral titers after IAV challenge of ferrets receiving antibiotic treatment. **a**–**d**, Ferrets (from SQ, PO and mock groups) were inoculated intranasally (i.n.) with 10^3^ PFU (**a** and **c**) or 10^6^ PFU (**b** and **d**) of A(H1N1)pdm09 virus 8 days after the second treatment with antibiotics or mock treatment. In **a** and **b**, the daily mean ± s.e.m. weight change from the preinoculation body weight is presented for all groups (*n* = 3). Two-way repeated-measures ANOVA: interaction *P* = 0.047, Dunnett’s multiple-comparisons test, time points D2 (*P* = 0.0374), D3 (*P* = 0.0071) and D4 (*P* = 0.0415), between PO (*n* = 3) and mock (*n* = 3) groups. In **c** and **d**, NW specimens were collected on alternate days p.i. and titered for the presence of infectious virus. Limit of detection, 10 PFU. Data are presented as the geometric mean with geometric s.d. Source data

**Fig. 4:**
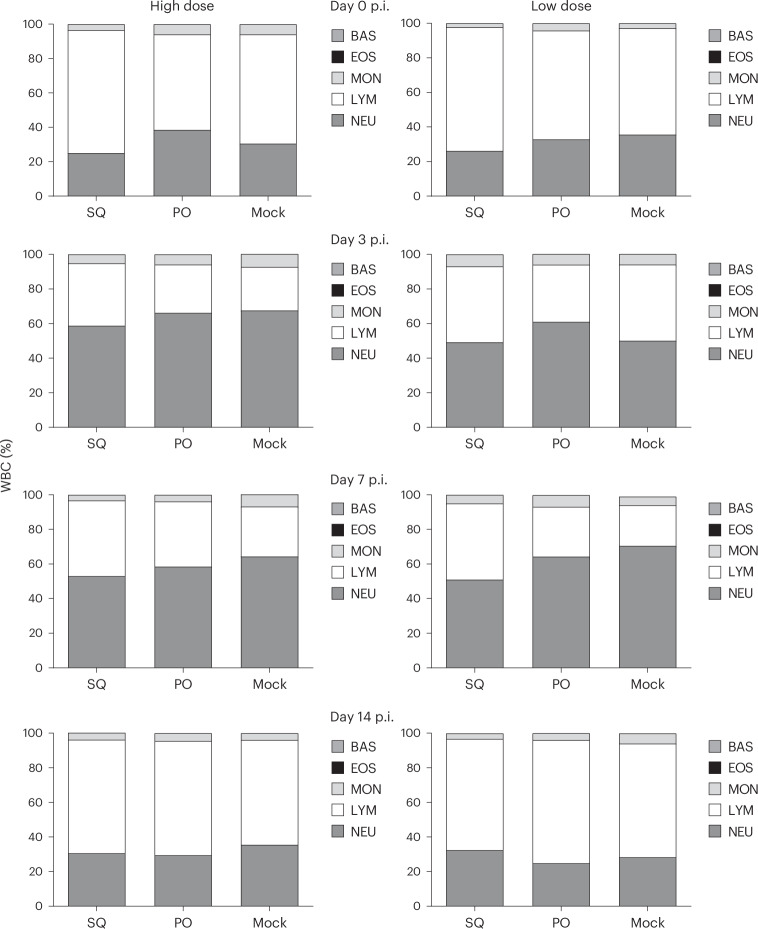
Kinetic analysis of circulating lymphocytes after influenza virus infection. Ferrets were inoculated i.n. with a high dose (10^6^ PFU) or low dose (10^3^ PFU) of A(H1N1)pdm09 virus 8 days after the second treatment with antibiotics (*n* = 3 per group). Blood was collected in EDTA vacuum phlebotomy tubes on the indicated days p.i. and analyzed on a hematology scanner. Mean average percentages of lymphocytes, neutrophils, basophils, monocytes and eosinophils in whole blood are shown. Source data

Ferrets inoculated with Neb/14 virus exhibited moderate clinical signs of infection independent of treatment administered or challenge dose; across all groups, mean peak weight loss ranged from 8.8% to 12.3% of preinoculation body weight, with a mean peak rise in body temperature from 0.9 °C to 1.5 °C observed (Fig. 3a,b and Table 3). During the acute phase of infection, ferrets that received either oral or injectable antibiotics exhibited a less severe weight loss compared with mock-treated animals (Fig. 3a,b). However, after viral clearance from the URT, animals that had received oral antibiotics had further decreased weight while most other animals had rebounded. Comparable frequency of respiratory signs (sneezing) was also noted across animals in all groups (data not shown). All groups exhibited transient but resolving lymphopenia (Fig. 4), with minor variation present between groups, typical of IAV challenge with A(H1N1)pdm09 viruses in this model^19,20^. Taken together, these findings support that antibiotic treatment led to generally similar outcomes compared with mock, but subtle group-specific alterations detected in clinical parameters were noted.

**Table 3:** IAV pathogenicity after antibiotic treatment of ferrets

Challenge dose^a^	Treatment group^b^	Weight loss^c^	Temperature^d^	Sneezing^e^	Peak NW^f^	Peak CW^f^	Peak RS^f^
High	SQ	11.2 (6–9)	1.4 (1–5)	3/3 (1–2)	8.2 ± 0.6	2.9 ± 0.8	2.6 (1/3)
	PO	11.6 (8–14)	0.9 (1–2)	3/3 (1–5)	7.8 ± 0.7	3.7 ± 0.9	1.9 ± 0.3 (2/3)
	Mock	11.7 (4)	0.9 (1–2)	3/3 (2–3)	7.7 ± 0.3	2.4 ± 0.6	1.5 (1/3)
Low	SQ	8.8 (6–10)	1.1 (2)	3/3 (2–5)	6.4 ± 0.3	2.7 ± 0.9	2.0 (1/3)
	PO	13.4 (7–14)	1.5 (2)	3/3 (1–5)	7.0 ± 0.5	3.8 ± 1.4 (2/3)	2.6 ± 0.7 (2/3)
	Mock	12.3 (5–8)	1.1 (2)	3/3 (2–4)	6.7 ± 0.4	2.6 ± 0.3 (2/3)	2.2 ± 0.4 (2/3)

^a^Ferrets inoculated i.n. with 10^6^ (high) or 10^3^ (low) PFU/mL of A/Nebraska/14/2018 A(H1N1)pdm09 IAV. ^b^Ferrets received treatment for 14 consecutive days (viral challenge day 8 post-treatment) via SQ injections of metronidazole, enrofloxacin and piperacillin–tazobactam, or oral feeding (PO) of metronidazole, enrofloxacin and amoxicillin–clavulanate acid. ^c^Percentage mean maximum weight loss days 1–14 p.i.; parentheses indicate day(s) on which maximum weight loss occurred. ^d^Mean maximum rise in temperature (°C) above baseline temperature (38.6–39.7 °C) days 1–5 p.i.; parentheses indicate day(s) on which maximum temperature rise occurred. ^e^The number of ferrets with observed sneezing days 2–12 p.i.; parentheses indicate the number of individual days each ferret was recorded sneezing. ^f^The maximum mean viral titer (recorded on days 1–5 for each animal in ferret NW, CW or RS specimens, expressed as log_10_ PFU/mL ± s.d. Parentheses specify the number of ferrets with infectious virus detected and reported when not 3/3 (limit of detection, 10 PFU/mL).

### Comparable IAV replication kinetics in ferrets independent of antibiotic treatment

We next assessed the role that commensal bacteria may contribute to viral shedding detectable in respiratory and extrapulmonary specimens collected from IAV-inoculated ferrets during the acute phase of infection. Kinetics and magnitude of IAV replication were assessed by measurement of infectious virus titers during the acute phase of infection and serologic titers from convalescent ferrets. All inoculated ferrets were productively infected with IAV, reaching mean peak titers in NW specimens across all treatment groups of ≥10^7.7^ PFU/mL and ≥10^6.4^ PFU/mL after high-dose and low-dose inoculation, respectively (Table 3). Viral titers remained consistently high through day 5 p.i. in all treatment groups before viral clearance (Fig. 3c,d). In agreement with prior work^21^, Neb/14 virus was detected in conjunctival wash (CW) specimens after intranasal inoculation, with comparable mean peak titers detected among both antibiotic-treated and control animals (Table 3); sporadic detection of infectious virus in rectal swab (RS) specimens was also noted across all treatment groups, at both challenge doses used. Comparable seroconversion to challenge virus was detected across all groups (hemagglutination inhibition titers 256–512). Taken together, these findings support that systemic antibiotic treatment in ferrets (before and after IAV inoculation) did not substantially alter the kinetics or magnitude of A(H1N1)pdm09 virus replication at either a high or low challenge dose in both respiratory and extrapulmonary specimens.

## Discussion

The ferret model represents an invaluable resource for risk assessment studies of viral pathogens that pose a threat to human health. While the majority of studies using this model utilize young, healthy and serologically naive animals^22^, there remains a need to evaluate viral pathogenesis in animals with altered health states and to better assess the diversity of bacterial species present in sites that support pathogen infection in preclinical models. Preclinical ferret models have been established with a wide range of health states and treatment administrations, including pregnancy^23^, obesity^24^, immunosuppression^25,26^, analgesics^27^ and others. Studies utilizing microbial DNA have identified a wide diversity of bacterial taxa present in the URT of outbred ferrets^13,14^ but have not specifically assessed the absolute abundance or possible reductions in culturable bacterial populations at this site. Our study extends this body of work by establishing and validating a ferret model of antibiotic treatment using two different delivery modalities that can be readily adopted by other institutions.

To improve reproducibility, ferrets used in this study were obtained from a single commercial vendor because the source of animals can represent an important source of variation^28^. Although there are limited recommendations for health monitoring of ferret colonies, textbooks that describe best practices for maintaining specific-pathogen-free health status are available^29^. Recent publications also shed light on the current state of knowledge about husbandry practices^30^ and host adaptations that may possibly be related to research applications^31^. Indeed, multiple commercial facilities make their colony health monitoring results publicly available, which is a testament to optimal animal biosecurity, an essential component for sound science. While there may be unrecognized differences between our vivarium and other laboratories, or even within our location across time, the IAV-infected ferret model is robust despite the statistical limitations on small sample sizes^4^.

As primary bacterial infections are not common in ferrets, and even less so in the respiratory tract, we recognize that our findings are less likely to be directly translated toward novel clinical cures and instead might be relevant for wider research areas. We purposefully used broad-spectrum antibiotics that target a wide spectrum of Gram-positive and Gram-negative bacteria in ferrets, to substantially reduce levels of bacteria compared with control animals, specifically at sites that support IAV replication in mammals. As the combined use of enrofloxacin, metronidazole and piperacillin–tazobactam successfully prevents secondary lung infection in CFTR-knockout ferrets^32^, we reasoned that these medications may penetrate URT tissues. Furthermore, we used pulsed therapy by administering all drugs once daily instead of multiple daily dosing. While such an approach takes advantage of the post-antibiotic effect of enrofloxacin^33^ and metronidazole^34^, it fails to meet steady-state concentrations that are ideal for β-lactams in general. While oral dosing may be more convenient per ease of delivery, the injectable route bypasses the digestive system that may pose limitations when treating ferrets with active illness.

We found that levels of cultivable bacteria were significantly reduced in NW specimens within 7 days of either antibiotic treatment administration, supporting the efficacy of both injection and oral delivery modalities dosed once daily. However, systemic or localized antibiotic treatment may not eliminate commensal bacteria from all anatomical sites, and culture-based methods may not detect all bacteria taxa that may contribute to health outcomes. While polymerase chain reaction-based methods have the potential to uncover additional organisms that are difficult to culture, they cannot distinguish live from dead microbes, and such distinction was essential to evaluate the effectiveness of our antibiotic regimens^35^. As this study was focused primarily on how the demands of bacterial colonization influences progression to disease, we did not specifically examine the restoration of commensal bacterial populations after antibiotic treatment that may occur following IAV inoculation, which has been shown previously in the ferret model^13^. Considerations for continued studies using long-term antibiotics should include the emergence of opportunistic infections with or without drug resistance, which was not directly tested here. Ultimately, the chosen route, frequency and duration should be evaluated by clinicians or researchers to ensure appropriate therapeutic dosing.

We elected to use two systemic routes that emulate antibiotic treatment courses utilized in humans; however, such an approach has been linked to unintended health consequences in mammals^18,36^. In this study, ferrets treated for 14-day intervals SQ or PO did not present with apparent clinical or hematological abnormalities relative to mock treatment that may have confounded viral challenge results. Yet, some ferrets within the same treatment group that received antibiotics via oral feeding experienced unusually progressive weight loss following viral clearance. One ferret was responsive to nutritional support, while another one reached the predetermined criteria for study termination. Although there were no gross abnormalities on the euthanized animal at necropsy, its remaining cage mate achieved considerable weight gain after the separation. Individual ferrets in the research and pet settings have been noted to dig food out of their bowls relentlessly, and this behavior trait may have contributed to the observed weight loss. Ultimately, we cannot exclude other possibilities that may be related to oral treatment and/or postviral effects. Further characterization of alterations in host immune responses, metabolism and tissue-specific pathology could uncover the mechanisms underlying the mild effects on weight loss observed between groups^37^.

Seasonal and zoonotic IAV can differ in their inherent virulence and can elicit different host responses in ferrets^38^. We propose that the antibiotic-treated ferret model presented here can be used to characterize microbial populations in the nasal passages before, during and after infection with seasonal or high-pathogenic influenza strains. As shown by infectious virus in CW and RS specimens in Table 3, selected strains are also capable of spread to extrapulmonary tissues in this species^9^, supporting the need to evaluate systemic host–microbe interactions in the context of antibiotic treatment, including collection of tissues both within and beyond the respiratory tract. While this study was limited to only A(H1N1)pdm09, further investigation of other IAV strains (such as zoonotic H1 subtype viruses, or other HA subtype viruses from avian and swine reservoirs) may reveal differential interactions with resident bacterial populations in the URT (and/or other sites) and warrants further study.

Beyond IAV, ferrets are a suitable model to study a broad range of viral pathogens, including those requiring manipulation in Animal Biosafety Level 3 (ABSL3) and ABSL4 settings^2^. Our assessment of both oral and injectable treatments, finding that both represent suitable delivery methodologies for antibiotic delivery in ferrets, provides options for adaptation of this model in environments where needle use may be discouraged. Beyond pathogenesis, ferrets are a valuable model to study the transmissibility of several viral pathogens, including but not limited to IAV^2^. Recent work has supported a role for bacteria in this process, as expelled aerosols containing infectious IAV and live bacteria have been detected from coinfected ferrets^8^, and antibiotic treatment to the upper airway has been shown to reduce subsequent IAV airborne transmission in the ferret model^14^. As this present study was limited to pathogenesis assessments, continued work exploring the transmission implications (including but not limited to IAV) in the context of antibiotic treatment is needed.

The severity of a primary influenza infection, in both seasonal and pandemic outbreaks, can be exacerbated by secondary bacterial infections^37^. As antibiotics are frequently prescribed to people who may have an active IAV infection to prevent sequela, there is a public health need to understand how reductions in commensal bacterial populations may modulate disease. Our findings support prior work showing that initial pathogenicity of a seasonal IAV is minimally altered during broad-spectrum antibiotic treatment. It should be noted that this cohort of ferrets did not harbor *Streptococcus pneumoniae* at detectable culturable levels, and secondary bacterial infections and/or pneumonia were not observed in mock-treated virus-infected ferrets throughout the acute or convalescent stages of observation, limiting our ability to assess whether reductions in the nasal bacterial microbiome may be associated with reductions in these potential clinical outcomes. However, the robust characterization of two antibiotic treatment modalities in ferrets presented here permits subsequent investigations to expand this work further, both in the context of viral infection and in other scenarios where antibiotic use is relevant and lacking.

## Methods

### Animals

Male, castrated and de-scented Fitch ferrets (*n* = 18; age, 12 months; weight, 1.0–1.5 kg; Triple F Farms) were used in this study. Hemagglutination inhibition assays confirmed that all animals were serologically negative to currently circulating influenza A(H1N1)pdm09, A(H3N2) and B viruses before the start of the experiment. Ferrets were pair-housed under conditions previously described^27^ within a Duo-Flo BioClean mobile environmental enclosure (Lab Products). Water and pelleted feed (Lab Diet FL14) was provided ad libitum. Enrichment was regularly provided in the forms of plastic play balls, toys on a metal chain, and edible paper cups. All described procedures were approved by the Centers for Disease Control and Prevention (CDC) Institutional Animal Care and Use Committee (3346BELFERC) and conducted in an AAALAC International-accredited vivarium at BSL2 conditions on a 12-h:12-h light:dark cycle.

### Study design

Baseline sampling and measurements were obtained before antibiotic treatment and throughout experimental infection on predetermined days. Ferrets were anesthetized for transponder placement, and all blood and specimen collections, virus inoculation and euthanasia as previously described^27^. In brief, anesthesia was conducted by intramuscular combined ketamine (10–30 mg/kg) and xylazine (2 mg/kg); euthanasia was performed by intracardiac administration of 1 mL/kg, 390 mg pentobarbital sodium and 50 mg phenytoin sodium per 100 mL. Administration of injectable antibiotics and measurements of temperature and weights were performed on conscious ferrets, unless anesthesia for sample collection was planned, in which case these procedures were conducted while ferrets were anesthetized. Oral antibiotics were always given to conscious ferrets. Temperatures were obtained via SQ temperature transponders that were placed in the dorsal space between the scapulae (via a 12-gauge applicator; 14 mm × 2 mm, IPTT-300, Bio Medic Data Systems (BMDS)). Observations of clinical signs (including lethargy, sneezing, nasal discharge and diarrhea during viral challenge), possible adverse events and other animal activities were performed cage-side before scheduled anesthesia or animal manipulation.

Ferrets were initially assigned into three groups of six animals each. The first group received three daily SQ injections (antibiotics specified in Table 1) in succession at distinct sites in 1 mL to 10 mL syringes using 25-gauge needles. Animals in the second group were syringe-fed (PO) daily (antibiotics specified in Table 1) with 3 mL oral syringes in succession containing suspensions mixed with meat-flavored baby food. All antibiotics were pharmaceutical grade and compounded when necessary. Animals in the final group (mock) received SQ injections with sterile saline and syringe feedings with only meat-flavored baby food. Ferrets were dosed daily for 14 days (first treatment), after which animals were held for a 9-week washout period, then redosed for 14 days as described above (second treatment). All ferrets were inoculated with IAV as described below on day 8 of antibiotic redosing.

### Specimen collection

NW, CW, RS and blood were collected from ferrets during initial antibiotic treatment and subsequent IAV challenge as previously described^27^. For bacteriology purposes, at least 400 µl of NW was transferred to a sterile 2-mL glass tube without additives immediately upon collection, sealed with a rubber stopper and maintained at 4 °C until culturing. For viral titration purposes, NW, CW and RS specimens were immediately placed on dry ice and stored at −70 °C until testing.

### IAV challenge and determination of viral load

A/Nebraska/14/2019 A(H1N1)pdm09 IAV was propagated in Madin-Darby canine kidney cells and titered by standard plaque assay as previously described^20,39^. Ferrets were inoculated intranasally (i.n.) with 10^6^ or 10^3^ PFU of virus diluted in 1 mL PBS (high and low doses, respectively). Weight and temperatures were recorded from all animals daily for 14 days p.i. Determination of viral load in NW, CW and RS specimens was performed by standard plaque assay as previously described^39^; the limit of detection was 10 PFU.

### Bacteriology

Culturing was performed on NW specimens by the Veterinary Diagnostic Laboratory (University of Georgia, Athens, GA, USA) approximately 12–16 h after collection. For aerobic culture, samples were inoculated into trypticase soy agar containing 5% sheep blood and incubated at 35 °C at 5% CO_2_ for 72 h. For anaerobic culture, samples were inoculated into thioglycolate broth and Brucella agar with 5% sheep blood and hemin–vitamin K and incubated at 35 °C under anaerobic conditions for 72 h. Gram-positive and Gram-negative bacteria were counted (minimum CFU/g) and morphologically distinct colonies were selected for subsequent identification using matrix-assisted laser desorption/ionization time-of-flight (MALDI-TOF) mass spectrometry.

### Hematological and serological assays

Blood for complete blood count analyses were collected in EDTA tubes and kept cold until analyzed the same day. CBCs were quantified using a VetScan HM5 analyzer per the manufacturer’s instructions. Blood for chemistry analyses was collected in lithium heparin tubes and performed using a VetScan VS2 chemistry analyzer and Comprehensive Diagnostic Profile rotors (Zoetis) per the manufacturer’s instructions. Two days before antibiotic dosing and 21 days p.i., serum was collected and tested for antibodies against homologous IAV via hemagglutination inhibition assay using 0.5% turkey erythrocytes as previously described^39^.

### Statistics

Data analysis was performed in Microsoft Excel, and statistical analysis was performed using GraphPad v8.4. For weight curves and bacterial culture samples that were collected over time and matched to individual animals, a two-way repeated-measures analysis of variance (ANOVA) was used, followed by Tukey’s multiple-comparison test. *P* values less than 0.05 were considered statistically significant.

### Reporting summary

Further information on research design is available in the Nature Portfolio Reporting Summary linked to this article.

## Online content

Any methods, additional references, Nature Portfolio reporting summaries, source data, extended data, supplementary information, acknowledgements, peer review information; details of author contributions and competing interests; and statements of data and code availability are available at 10.1038/s41684-025-01574-9.

## Supplementary information

Supplementary Information Supplementary Table 1.

Reporting Summary

Supplementary Data 1 ARRIVE checklist.

## Source data

Source Data Fig. 1 Per-ferret CFU counts.

Source Data Fig. 2 Per-ferret blood chemistry results.

Source Data Fig. 3 Per-ferret normalized ferret weights and viral titers.

Source Data Fig. 4 Per-group percentages of WBCs in whole blood.

## Data Availability

The data that support the findings of this study are presented in the Supplementary Information and are available from the corresponding author upon reasonable request. Source data are provided with this paper.
