# Supplementary material for: Establishment and validation of a ferret model for systemic antibiotic treatment during influenza A virus infection
Source: Lab Anim (NY). 2025 Jun 19;54(7):188–94. doi: 10.1038/s41684-025-01574-9 (PMC12213610; doi:10.1038/s41684-025-01574-9)
Supplement: Supplementary file 1 — Supplementary Table 1. [file 41684_2025_1574_MOESM1_ESM.pdf]

lab animal

Supplementary information

https://doi.org/10.1038/s41684-025-01574-9

Establishment and validation of a ferret model for systemic antibiotic treatment during influenza A virus infection

In the format provided by the authors and unedited

Supplementary Table 1. Blood chemistry analyses of ferrets pre- and post-14 day antibiotic treatment.

Serum chemistry[a]	Days post treatment	Treatment route: Oral	Treatment route: Inject
ALB (g/dL)	0	4.20 ± 0.13	4.47 ± 0.36
	14	3.82 ± 0.29	3.87 ± 0.22
ALP (U/L)	0	36.60 ± 5.64	29.00 ± 13.27
	14	28.00 ± 5.59	30.20 ± 7.95
ALT (U/L)	0	219.33 ± 108.99	124.00 ± 41.22
	14	144.50 ± 68.41	93.17 ± 39.06
AMY (U/L)	0	22.80 ± 2.68	16.20 ± 5.50
	14	18.33 ± 2.80	13.17 ± 7.31
TBIL (mg/dL)	0	0.27 ± 0.05	0.22 ± 0.08
	14	0.25 ± 0.05	0.28 ± 0.04
BUN (mg/dL)	0	27.00 ± 10.02	38.00 ± 3.69
	14	16.00 ± 3.10	22.17 ± 4.17
CA (mg/dL)	0	9.50 ± 0.15	9.88 ± 0.37
	14	9.38 ± 0.25	9.62 ± 0.28
PHOS (mg/dL)	0	6.47 ± 0.57	7.30 ± 0.72
	14	5.55 ± 0.34	6.03 ± 0.62
CRE (mg/dL)	0	0.55 ± 0.22	0.63 ± 0.27
	14	0.44 ± 0.09	0.45 ± 0.15
GLU (mg/dL)	0	117.33 ± 36.86	111.50 ± 14.64
	14	115.50 ± 22.93	119.67 ± 21.87
NA+ (mmol/L)	0	149.33 ± 1.97	153.17 ± 1.60
	14	148.00 ± 1.41	148.33 ± 1.75
K+ (mmol/L)	0	5.10 ± 0.09	5.10 ± 0.14
	14	4.92 ± 0.30	5.16 ± 0.24
TP (g/dL)	0	5.85 ± 0.21	6.28 ± 0.52
	14	5.32 ± 0.41	5.65 ± 0.41
GLOB (g/dL)	0	1.65 ± 0.23	1.80 ± 0.14
	14	1.52 ± 0.25	1.78 ± 0.24

[a] ALB, albumin; ALP, alkaline phosphatase; ALT, alanine aminotransferase; AMY, amylase; TBIL, total bilirubin; BUN, blood urea nitrogen; CA, calcium; PHOS, phosphorus; CRE, creatinine; GLU, glucose; NA+, sodium; K+, potassium; TP, total protein; GLOB, globulin. All levels are reported as the mean ± standard deviation of n=6 animals per group.
